# The Real Attitude of the Dental Specialty toward Medicine

**Published:** 1902-05-15

**Authors:** Frank W. Sage

**Affiliations:** Cincinnati


### THE REAL ATTITUDE OF THE DENTAL SPECIALTY TOWARD MEDICINE.

BY FRANK W. SAGE, D.D.S., CINCINNATI.

Read before the Cincinnati Odontological Society.

Before the recognition of dentistry as a specialty of medicine in the year 1892, thoughtful, ambitious men in the dental profession had long been turning this way and that, with expressions of intense yearning for relief from restrictions felt to be humiliating to an honorable profession. In dental meetings the question kept recurring, "Is dentistry a profession?" It was as if the dentists, having organized themselves into a nameless *something*, and having arrogated to that something the name "profession," had come to doubt whether fact and public sentiment sustained them in the position assumed. There was a half-appealing attitude toward the medical profession for assistance in raising dentistry to a proper level. Frankly expressed, this appeal might have been worded thus—"What can we do to make our poor, nondescript, half-fledged profession respectable, and ourselves, its members, respected?" This refrain sounded throughout every discussion in the dental societies. Whether the theme discussed was the education of the public—that ignis fatuus of the dental convention—the relation of dentistry to medicine, or the preparation of the dental matriculate, the same familiar plaint constantly recurred. At the same time the attitude toward the medical profession was half defiant. It was the fashion for members who could think of nothing else to say, to arraign the physician for his presumed ignorance of dental science.

When recognition came, however, all this was instantly changed. A few independent spirits indeed were for repudiating the proffered honor, holding that dentistry had given as much to medicine as it had received. In the end, however, they waived their objections and joined in the general congratulations. Dentists everywhere felt an accession of dignity which took little account of the fact that in recognizing dentistry as a specialty of medicine the Medical Association in session at Washington had not proclaimed all dentists to be medical specialists.

While the result of that recognition was to greatly stimulate the dental profession, inducing many already in practice to take the medical degree, a large number of intelligent, capable men in the dental profession maintained that dentistry is nevertheless a distinct profession, developing its peculiar resources along lines which rarely, or at least only in a measure, converge toward those on which medicine progresses, and that it serves its patrons in ways and by means wholly foreign to those physicians employ. To the present day this view prevails with not a few. The situation is peculiar, unprecedented, significant of an anomalous relationship of dentistry to the parent stem, curious and interesting so the last degree. How did this come about?

The dental specialty, springing originally from medicine, as did all other medical specialties, seems to have required to be grafted upon another stock, or, to extend the figure, to be even transplanted to another bed supplying nourishment of a description wholly different from that which served for the development of the other medical specialties. In one or two respects only the dentist's service to his patient resembles that of the physician or the surgeon. He may exhibit remedies or employ surgical interference, but this office may be merely preliminary to a peculiar service which is to follow, something wholly extraneous to medical science, while the physician's ministrations begin and end with a prescription. Herein is dentistry unique. No other specialty of medicine furnishes its counterpart. No other specialty could have so developed along lines of its own selection, appropriating empirically such principles of practice and such remedies as suited its purpose, and yet finally attain a success practically untainted by empiricism. In earlier days the dentists, perplexed and unable to solve pathologic or therapeutic problems, either stumbled helplessly over them or passed by on the other side, finding them after all not absolutely essential. What knowledge the dentist acquired of materia medica, pathology, and other branches of medical science, he got by inductive processes, formulating no principles covering classes of cases in practice. Yet despite these drawbacks of ignorance, the science and art of dentistry have finally rounded out to the proportions of a legitimate specialty of medicine. What less than a miracle is this?

Conceive if you can of the oculist attaining to perfection of skill after the manner in which the dentist of seventy or eighty years ago set about to learn dentistry. Imagine a man dropping his carpenter's plane to enter the office of an oculist of acknowledged skill, with a view of learning to be an oculist. He buys a copy of "The Principles and Practice of Optical Science," into which he occasionally dips, when in difficulty. His main reliance for information, however, is in watching his "preceptor" and imitating his processes. We see him remove crystallin lenses for patients, and retinas, and we observe that the patients go away evincing no consciousness of hav'ng suffered irreparable loss, but rather seem joyous over their relief from pain. Now we see our student working hours fashioning glass eyes, which later his "preceptor" inserts for a waiting patient. We hear him say, "You must persevere in using these, and before long you will be able to see with them quite as well, if not better, than you ever could with your natural eyes." Later we see the preceptor operating hour after hour for another patient, repairing a natural organ. "You should remember, sir, that one natural eye is worth a dozen glass ones," he replies to the patient's remonstrance against the exorbitant fee. Such it seems is the duplicity of oculists!

Now further picture the student after two or three year's instruction under this able teacher pursuing practice on his own account, year after year accumulating fame and honors. Failures he occasionally has, but no death is ever charged to him, no whisper of malpractice ever assails him. This picture, preposterous though it seems, needs only slight retouching to pass for a faithful presentment of the dentist's schooling not so long ago. It must appear that the oculist could never so much as make a start in the direction of success unless he had first traversed the entire field of medical science. He must be competent at the outset to trace from symptoms disclosed in the eye such disorders as syphilis, paralysis, locomotor ataxia, the various anemiae, and other diseases. His patient knows without special prompting that irreparable loss may follow the slightest error of diagnosis or treatment, while the popular estimate of the dentist's responsibility is expressed in a wide-spread belief that he can fall into no error which a pair of forceps deftly wielded will not correct.

It appears then that the comparative unimportance of the dentist's service, for one thing, accounts for the fact that dentistry has so long been left to its own resources, but an element liable to be overlooked has had much to do with the dentist's confining himself to so comparatively narrow a field. It is the time required for the service rendered. While the oculist may wait on ten to a hundred patients daily, and still spare time for hospital work or the lecture room, the dentist may be all day long treating and filling two or three teeth for a single patient.

Here then are facts which seem to account for the present attitude of dentistry toward medicine; an attitude not lacking in friendliness, yet expressing, as many believe, a feeling of incompatibility. The dentist who is of the fibre to be a leader among dentists is essentially mechanical in his instincts. He may possess the instincts of the physician as well, but his bent is mechanical. Whatever is to be done for improving the status of the coming dentist will fail of its purpose if it be not kept strictly in mind that he must remain a dentist. It is possible that the question of affiliation with the medical faculty must be relegated to the rear, in considering what the crying need of dentistry actually is. No mere sentiment referring to the elevation of the profession by any factitious means is to be considered. We can not dignify dentistry by making it over into something else, even though that something else be medicine. Dentistry needs no dignifying. Its individual members through their individual personal influence confer upon it or withhold from it the only dignity worthy of the name. The public cares nothing for us as a profession; it takes cognizance of us only as individuals, or as groups of individuals, and chooses from among us such as it deems best qualified to minister to its peculiar needs. But the age of superstition is not fully past; the occult in medicine has had much to do with the physician's being esteemed above the dentist. Even that, however, is passing; so great a man as the lawyer, once supposed to outrank all other professional men, has come to be irreverently spoken of and to have his fees criticised.

The true standard of respectability in any profession is an individual standard. The dentist who is by nature, education and breeding worthy of the respect of his patrons usually finds no occasion to lament that his profession is not respectable. He may regret moving along a narrow path, but he has no vain yearnings after the merely adventitious aid of a title. The attitude of dentistry toward the medical specialty is a composite of the attitudes of its thousands of individual members. One dentist expresses in his attitude a genuine desire to know more of medicine in order that he may do more and better for his patients. A second is impelled by pride to seek a more honorable degree than that of D.D.S., having a regard for the prestige the title may confer. A third cares nothing for the mother science. A fourth is openly hostile to the suggestion that dentistry needs any assistance from the medical profession. This composite picture requires to be studied with relation to any and all suggestions for improving the status of dentistry and the dentist. We may rule incompetent men out of the profession, but we can not rule out temperament or natural aptitude. We can not coerce men who have a taste for dentistry and none for medicine into the medical fold. These matters the dental college must deal with, for the medical college could not supply to its students a knowledge of dental science, to say nothing of a knowledge of dental art, if the dental college were abolished. Dentistry must be greatly improved, both as to its science and its art. The picture of the rise and development of dentistry portrays after all only a sorry success. The mischief wrought by unscientific men during those long years of development—who can tell!

The history of all important reforms shows that the exciting influence comes from without the circle of the distinct organization, whatever it may be, but the active agencies by which the reform is set afoot spring up from within the organization. Granting that this picture of the attitude of dentistry toward medicine is a fair presentation of facts, there remains still to be considered the attitude of the public toward both professions. The dentist's patrons are satisfied with his services so long only as they believe them to be the best the science and art of dentistry are capable of supplying, but as a matter of fact they find so much of imperfection in his ministrations that the question of the why and wherefore of failure is constantly being suggested to him. So that he in turn is constantly asking, " How can I

improve my services to my patrons?" That is if he is really possessed of the progressive spirit. Which finally brings us face to face with the question, whether the great mass of the profession, including the progressive and non-progressive, are likely to be considered in any proposed measures for improving the dentist's status. Foremost of all questions is, what is the first that can be done for the education of the number of dentists adequate to the popular demand for dentists? It is not perhaps an unfair assumption that the leaders of our profession, those who have done most for the science and art of dentistry, should be the only arbiters of this question. It can not be delegated to the medical profession to prescribe boundaries and determine final principles on which the coming dentist's education shall be conducted. That falls inevitably to the lot of the dental profession.

DISCUSSION.

Dr. J. S. Cassidy: The high standing of medical men is not due to any superior knowledge but to the mystery surrounding the remedies and treatment. In early days medical science was far from perfect—fever patients were not allowed water to drink, the sick were not given the food which they required, blood-letting was constantly resorted to, and the methods of treatment were all wrong. I have always believed that a dentist could properly cover his field without going through a medical school. Nevertheless, the more a man knows of medicine the better equipped he is. For instance, in order to give anesthetics intelligently he must know something of physiology and pathology. We can not consult with physicians regarding diseases of the teeth, as they are for the most part absolutely ignorant on this subject. I have had physicians come to me and ask to have pulps killed in abscessed teeth. Medical men will frequently treat a dental case for years which a dentist could relieve in ten minutes. How often have physicians and surgeons cauterized for months a running sore, due to a dead pulp in a tooth, which has healed spontaneously after treatment from a good dentist.

Dr. C. M. Wright: The late Dr. McKellops was one of those who was opposed to accepting the honorary degree from a medical college. For my part, it does not matter whether physicians acknowledge us as specialists or not, since it is patent to all observers that along certain lines we are one and the same. I agree with the essayist that the respectability of the profession is in the hands of its individual members.

Dr. T. I. Way: One disreputable member of the profession can do more to injure us in the opinion of the public than the example of a dozen reputable practitioners can do to help us.

Dr. M. H. Fletcher: Of all professional men the dentist needs the best equipment for practice, and he must have a medical education in order that he may know systemic conditions, temperament, etc. Without it he may not even succeed in that work which some insist is the principal and sole function of the dentist. At present the dental colleges do not teach pathology thoroughly enough. It is true that individual dentists make their own standards of respectability, but it is also true that the public estimates us according to long recognized standards.

Dr. A. A. Kumler: With a large number of the profession the distraction of studying two sciences and the dividing of individual energy would result in a lessening of the dentist's ability.

Dr. O. N. Heise: It is the individual in dentistry who has established the standard by which the public judges us, which fact gives a peculiar emphasis to the assertion that the dentist must know more in order to be rated higher, for as a dentist he ranks only as a dentist. The important thing is not that he acquire the medical degree, but that he shall be able to manage a case requiring medical knowledge and experience just as well as the physician, for it is seriously to the disadvantage of the dentist that he is ever forced to the necessity of calling in a physician. Dentists need more thorough education, and I do not believe a first-class dental college compares with a first-class medical college in teaching pathology, physiology, materia medica, etc.

Dr. C. M. Wright: I must protest against the statements of Drs. Fletcher and Heise, that general pathology, physiology, etc., are taught more thoroughly in a medical than a dental school. I teach general—not special—pathology, in a dental college, and find that graduates from medical schools, placed side by side with dental students, rank second to them. Several years ago I refused the degree that these "M.D., D.D.S." men appreciate too highly, because I believed that a dentist need not be ashamed of his calling; furthermore, none of my medical friends would think any more of me for having the title of M.D. These dentists who also hold the degree of M.D. (Mystical Doctor), try to impose on us plain D.D.S,'s and pose as something higher and better. I never advise a dentist to try for the M.D. degree. It spoils him. There is a tendency developed toward encephalic hypertrophy.

Dr. J. N. Crouse, editorial in *Dental Digest:* Personally, we would hold a middle view. The dentist is primarily a mechanic, and a student with mechanical genius and a fair education will make a better dentist, in the general acceptance of the term, than a student utterly lacking in mechanical talent, but possessing a broad, thorough education. We do not mean by this that students with only mechanical ability should be admitted to our dental colleges, for that would soon bring dentistry to the level of a trade pure and simple. On the other hand, we do not believe students who have only a fine education should be accepted, as that would make us a class of theorists. The fact that there are to-day over one hundred dental laboratories throughout the country, doing all the mechanical work for a large number of dentists, is a sad commentary on the mechanical training or ability of the average practitioner.

First of all the student must have mechanical ability or he can not practice dentistry, but it is just as essential that he possess a thorough general education and have a mind trained by study and observation. Otherwise, he is fit for nothing but the purely mechanical work and can not rank as a dentist. If a student has these two qualifications, mechanical and intellectual, he should be able to obtain in the dental college all the knowledge of general medicine which he will be apt to need, and we believe all the first-class schools to-day give him that opportunity if he will embrace it. While we would urge students to take a medical course before entering a dental college, if they have the time, money and desire to do so, we would recommend it for its broadening and deepening influences, and not because we think a man must be a physician first and a dentist next. This latter seems to be the view held in Europe, and as a consequence of the exaltation of general medicine above the dental specialty, the mass of the people over there suffer from lack of any dental service whatever.

However, dentistry in this country seems to be working out its own salvation, and if the dental colleges extend their courses of study to meet the requirements made upon a dentist in active practice, and see to it that only well-balanced students, those possessing both mechanical skill and a broad education, are admitted, we need have no fear that the reputation of our profession will suffer. The colleges deserve all credit for their continued efforts to raise the standard, and we think the matter can be safely left in their hands.—*Digest.*

